# Characterization of Hepatitis B virus (HBV) genotypes in patients from Rondônia, Brazil

**DOI:** 10.1186/1743-422X-7-315

**Published:** 2010-11-12

**Authors:** Alcione O Santos, Mónica V Alvarado-Mora, Lívia Botelho, Deusilene S Vieira, João R Rebello Pinho, Flair J Carrilho, Eduardo R Honda, Juan M Salcedo

**Affiliations:** 1 Research Center for Tropical Medicine - CEPEM/Tropical Pathology Research Institute-IPEPATRO. Porto Velho, RO, Brazil; 2 Laboratory of Gastroenterology and Hepatology, São Paulo Institute of Tropical Medicine and Department of Gastroenterology, School of Medicine, University of São Paulo, São Paulo SP, Brazil

## Abstract

**Background:**

Hepatitis B virus (HBV) can be classified into nine genotypes (A-I) defined by sequence divergence of more than 8% based on the complete genome. This study aims to identify the genotypic distribution of HBV in 40 HBsAg-positive patients from Rondônia, Brazil. A fragment of 1306 bp partially comprising surface and polymerase overlapping genes was amplified by PCR. Amplified DNA was purified and sequenced. Amplified DNA was purified and sequenced on an ABI PRISM^® ^377 Automatic Sequencer (Applied Biosystems, Foster City, CA, USA). The obtained sequences were aligned with reference sequences obtained from the GenBank using Clustal X software and then edited with Se-Al software. Phylogenetic analyses were conducted by the Markov Chain Monte Carlo (MCMC) approach using BEAST v.1.5.3.

**Results:**

The subgenotypes distribution was A1 (37.1%), D3 (22.8%), F2a (20.0%), D4 (17.1%) and D2 (2.8%).

**Conclusions:**

These results for the first HBV genotypic characterization in Rondônia state are consistent with other studies in Brazil, showing the presence of several HBV genotypes that reflects the mixed origin of the population, involving descendants from Native Americans, Europeans, and Africans.

## Background

Human hepatitis B virus (HBV), which is the prototype member of the family *Hepadnaviridae*, is a circular, partially double stranded DNA virus of approximately 3200 nt [1]. This highly compact genome contains four major open reading frames encoding the envelope (preS1, preS2 and surface antigen - HBsAg), polymerase (HBPol) and X (HBx) proteins [2]. HBV infection is a relevant global health problem, with 2 billion people infected worldwide, including 350 million of them suffering from chronic HBV infection. HBV infection results in 500,000 to 1.2 million deaths per year caused by chronic hepatitis, cirrhosis, and hepatocellular carcinoma and is the 10^th ^leading cause of death worldwide [3]. The mechanisms for persistent HBV infection are not fully understood, but they seem to involve several aspects, including genetic components [4]. The role of genetics components of the virus and the host in the natural history of hepatitis B including HBV genotypes and subgenotypes; basal core promoter and pre core mutations; HBV DNA serum levels and co-infection with other viruses (particularly hepatitis C and human immunodefficiency viruses) have been recently reviewed [5].

HBV has been classified into nine different genotypes, designated from A to I [6], they that represent genetically stable viral populations that share a common, separate evolutionary history. They emerged in specific human populations and migrated with their hosts to other areas in the world, leading to their present geographical distribution [7]. Genotype A is distributed globally and is the main genotype found in Europe, North America, Africa and India. Genotypes B and C are predominant in East and Southeast Asia [8]. Genotype D is mainly found in the Middle East and Mediterranean countries but it has been reported globally, whereas genotype E seems to be predominant in western-sub-Saharan Africa [9,10]. HBV/E has not been found outside Africa, except for a few rare cases mostly in individuals with an African background. Nevertheless, it was recently reported the presence of this genotype in a specific community in Colombia [11] and in the north of India [12]. Genotype G has been characterized in samples from USA, Mexico and France and appears primarily to be present as a coinfection with another HBV genotypes, most commonly genotype A. Genotypes F and H are found almost exclusively in Central and South America [13,14]. Recently, HBV genotype I was described in northwestern China, Vietnam and Laos [6,15,16].

Most genotypes have been divided into subgenotypes with distinct virological and epidemiological properties. In addition, recombination among HBV genotypes increases the viral variability itself [17].

Genotype A is subdivided into seven subgenotypes (A1 to A7) [18,19]. Isolates belonging to subgroup A1 have been mostly identified in African populations and their descendants [20-22]. Subgenotype A2 is mainly found among Europeans, whereas subgenotype A3 has been identified in Central and West Africa [23,24]. Subgenotype A4 was reported in Gambia [18,22] and subgenotype A5 was reported in Nigeria and among African descendants in Haiti [25]. Subgenotype A6 includes strains from African-Belgian patients from Congo and Rwanda [26] and A7 was found in Rwanda and Cameroon [19].

Genotype D was previously divided in 4 subgenotypes (D1 - D4) [27] found in different continents, spreading particularly around the Mediterranean Basin to the Asian continent. New subgenotypes, D5 to D7 were later described in India [28], Indonesia [29], and in the Mediterranean Basin [30], respectively.

Genotypes E and G are not subdivided in subgenotypes [31,32]. Genotypes F and H are the ''New World'' genotypes found in indigenous populations from Alaska to Central and South America. Genotype F is divided into 4 subgenotypes: F1-F4. Subgenotypes F1 and F2 have been further divided in F1a, F1b, F2a and F2b [14,33-35]. Genotype H is very closely related to genotype F and was initially thought to be a clade of genotype F [13,36].

The state of Rondônia is located in the Southwest of Brazilian Amazon and borders with other Brazilian states (Mato Grosso - East, Amazonas - North, Acre - West) and Bolivia (West and South). Currently, it is not clear the general prevalence of HBV in Rondônia state. Katsuragawa et al., [37] found frequencies of 44.5% for anti-HBc and 6.7% for HBsAg studying the serologic markers of hepatitis B and C among the inhabitants of the upper Madeira river, between the localities of Santo Antonio and Abunã, in the Municipality of Porto Velho, Rondônia.

In the last Brazilian census, carried out in 2000, there were 1,380,952 inhabitants in Rondônia State, with the following ethnic background: European-descendants - 588,568 (42.62%); African-descendants - 63,452 (4.59%); Asian-descendants - 3,094 (0.22%); mixed - 698,309 (50.56%); Indigenous people - 10,683 (0.77%); not known - 16,846 (1.22%) [38].

The aims of the present study were to characterize the HBV genotypes circulating in Rondônia state, Brazil, and to infer about their origin using phylogenetical analyses approaches.

## Methods

**Figure 1:**
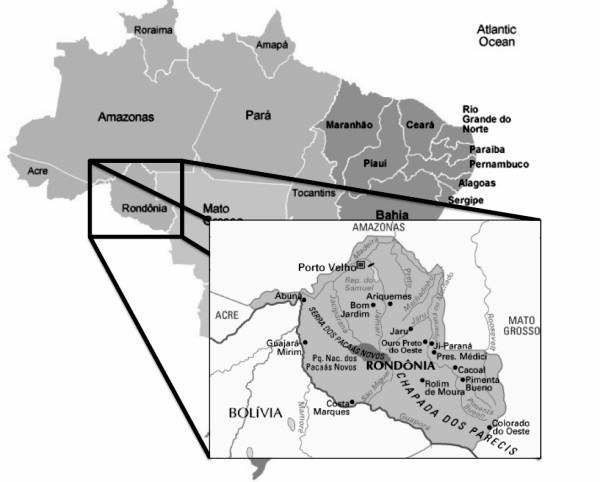
**Geographic location of Rondônia state, Brazil**.

This study was carried out in the state of Rondônia, Brazil (Figure 1) and included 40 serum samples from patients chronically infected with HBV. Of these 40 patients, 26 (65%) were asymptomatic and 27 (67.5%) did not have liver cirrhosis. All samples are HBsAg positive for at least six months, but only 26 (65%) of them were also HBeAg positive, as previously determined by routine serological assays during patients follow up. Patients had between 18 and 60 years old (mean age: 30 years); sex distribution was 28 (69%) men and 12 (31%) women and all of them were under medical assistance at the Research Center for Tropical Medicine (CEPEM), in Rondônia. Indigenous people patients, pregnant women and patients with other associated chronic diseases were excluded from the present analysis.

For the viral DNA extraction from 200 μl serum, it was used QIAamp DNA Mini Kit (Qiagen, Germany) according to the manufacturer's standards. The precipitated DNA was resuspended in 200 μl of elution buffer and stored at -20°C until use.

To avoid false-positive results, strict procedures proposed for nucleic acid amplification diagnostic techniques were followed [39]. Samples were first amplified with primers previously described [40] in order to get a 416 base pairs (bp) fragment partially covering the HBsAg coding region (S). A fragment of 1306 bp partially comprising HBsAg and Polymerase coding regions (S/POL) was then amplified from the samples that had been positive in the previous step [13].

Amplified DNA was purified using ChargeSwitch^® ^PCR Clean-Up kit (Invitrogen, São Paulo, Brazil). Sequencing was performed in an ABI Prism^® ^377 Automatic Sequencer (Applied Biosystems, Foster City, CA, USA) [41] using dideoxy nucleoside triphosphates (ddNTPs) containing fluorescent markers (Big Dye^® ^Terminator v3.1 Cycle Sequencing Ready Reaction kit - Applied Biosystems, Foster City, CA, USA).

The quality of each electropherogram was evaluated using the Phred-Phrap software [42,43] and consensus sequences were obtained by alignment of both sequenced strands using CAP3 software available at the web page Eletropherogram quality analysis http://asparagin.cenargen.embrapa.br/phph/.

Initially, sequences obtained in this study were genotyped by phylogenetic reconstructions using reference sequences from each HBV genotype obtained from the GenBank (n = 383) (data available upon request). These sequences comprising partial HBsAg and Polymerase coding regions (S/POL) were aligned using Clustal X software [44] and edited in the SE-AL software (available at http://tree.bio.ed.ac.uk/software/seal/). For the phylogenetic analysis, the missing nucleotides were coded as "missing characters" in nexus block. Bayesian phylogenetic analyses were through by Markov Chain Monte Carlo simulation implemented in BEAST v.1.5.3 [45] ten million generations were sufficient to obtain the convergence of parameters. The analyses were performed under relaxed uncorrelated lognormal molecular clock using the model of nucleotide substitution (GTR+G+I) obtained previously by Modeltest v3.7 [46]. The maximum clade credibility (MCC) tree was obtained from summarizing the 10,000 substitution trees and then it was removed 10% of burn-in using Tree Annotator v.1.5.3 [45].

## Results and Discussion

**Figure 2:**
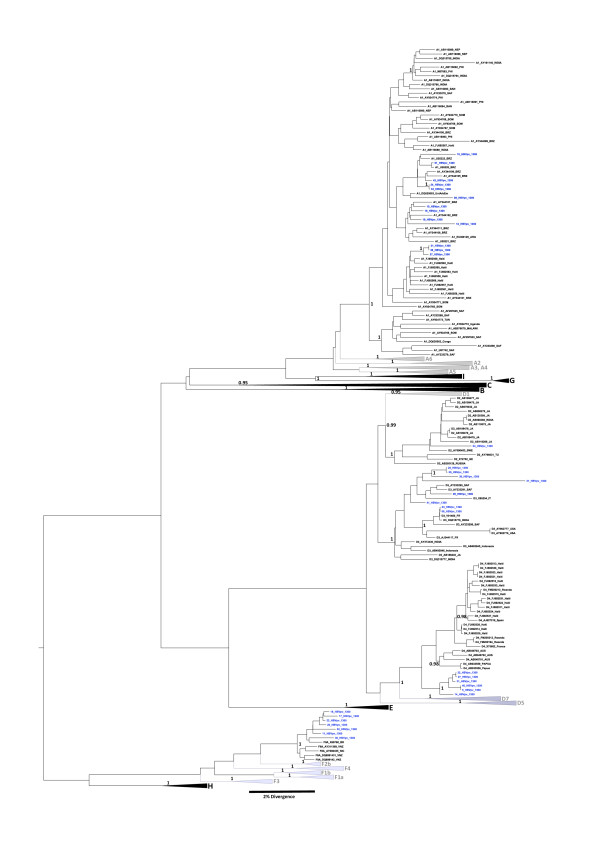
**The maximum clade credibility (MCC) tree was estimated by Bayesian analysis of 383 S/POL sequences with 1306 bp of Hepatitis B virus strains**. The posterior probabilities ( > 0.95) of the key nodes are depicted above the respective nodes. The HBV isolates from Rondônia state are represented in blue and were analyzed together other worldwide strains. The collapsed clades correspond to the other genotypes of HBV.

PCR for the S/POL region (1306 bp) was performed in all the 40 samples and 35 of them showed positive results (Figure 2). The HBV genotypes distribution found was: A (37.1%), D (42.8%), F (20.0%), while up to the subgenotype level we found A1 (37.1%), D3 (22.8%), F2a (20.0%), D4 (17.1%) and D2 (2.8%). Sequences were deposed in the GenBank at accession numbers: HM101096 - HM101130.

HBV/A1 samples from Rondônia state did not cluster together in a single group in the phylogenetic tree and only few sequences remained close to previously reported Brazilian sequences. Subgenotypes D3 and F2a showed the same pattern. These results suggest that probably several different entries of these HBV subgenotypes occurred in this state. On the other hand, genotype D4 sequences clustered in a single group. Nevertheless, as there are few reported sequences from this subgenotype [25,47,48], it was not possible to robustly infer the entry pattern for this subgenotype.

This is the first study reporting the HBV genotypes in Rondônia state, Brazil. A molecular characterization of HBV sequences is important in establishing the evolutionary origins and patterns for viral dispersal. Several reports previously determined the preponderance of genotypes A, D and F in South America [40,49-53]. This finding agrees with the origins of Brazilian population, which is a mixture of European-descendants, Indigenous people and African-descendants.

Previous studies have shown that genotype A was the most frequent in different Brazilian populations [21,40,53-55]. Recently, this genotype was found in 75% patients from Rio de Janeiro [56]. Most of these cases belonged to subgenotype A1, which is the same that was detected in Rondônia. Genotype A was also found among HBV carriers in the state of Acre, which borders the state of Rondônia, in 25 (73.5%) of 34 HBV carriers [57].

Subgenotype A1 was related to the presence of isolated communities of African-descendants, as recently reported in Mato Grosso do Sul State, Central Brazil [58]. It is estimated that about 3.5 million Africans arrived in Brazil in the period between 1551 and 1850 [59]. Currently, there are over 1,000 communities officially identified as remnants of Quilombo, the Brazilian name for small isolated communities made from runaway-slaves where their descendants lived in communities since the slavery period [58,60]. Most of the African-descendants currently living in Rondônia came for the construction of the Madeira-Mamoré Railway, a hallmark in Rondônia state history, that was built by many African-descendants workers in the beginning of twentieth century. Most of them had come from the Caribbean Barbados in a different context from most of the slaves that came directly from Africa [61]. Studies analyzing HBV genotypes in Barbados should be carried to allow a better comparison among Rondônia and Barbados circulating virus. Based on the phylogenetic analysis, as the different sequences from Rondônia are interspersed in the tree and clustered together with other Brazilian sequences (that mostly come from Rio de Janeiro State), as well as with Haitians sequences in another branch, we suggest that subgenotype A1 had different entries in Rondônia, i.e., different viruses were the founders of this population.

Genotype D predominates in the Mediterranean area [62]. Subgenotype D1 occurs mostly in the Mediterranean basin and Middle East. D2 has been reported in India, Japan, Europe and the United States [63]. D3 was found in South Africa, Brazil, Rwanda, Costa Rica and the United States. Finally, D4 was reported in Australia, South Africa, Somalia, Rwanda and Oceania [27,47,48]. In this study, genotype D was prevalent (42.8%) and its subgenotypes were D2, D3 and D4. Since the number of sequences obtained for each HBV/D subgenotype found in Rondônia was small, it was not possible to infer about the origin for each one.

In all the three states located in Southern Brazil, HBV genotype D was previously detected: Paraná [64], Santa Catarina [65] and Rio Grande do Sul [66]. Genotype D is the most frequent in Southern Brazil, whereas genotype A is the most frequent in all other regions [67,68]. In Italy, genotype D is largely the most frequent and is found in 73 to 80% of the patients infected with HBV [69,70]. The Italian government claimed that there are 25 million Brazilians of Italian ancestry, which would comprise the largest population with Italian background outside Italy. During the last quarter of the nineteenth century, several Italians were stimulated to migrate to Brazil and other countries, such as Argentina and the United States. Italians migrants settled down mostly in Southeast (São Paulo state) and South Brazil (Paraná, Santa Catarina and Rio Grande do Sul states). South Region inhabitants latter migrate to Center West and Amazon states, including Rondônia. A deeper characterization of hepatitis B virus genotypes found in Southern Brazil is needed to better understand the migration of hepatitis B virus genotypes in Brazil, particularly for genotype D.

Genotype F is the most divergent and considered indigenous in the Americas. Mello *et al. *[55] showed that genotype F had a low prevalence in Brazil. All genotype F sequences here described belonged to subgenotype F2a, that it is the same that is found in other Brazilian and Venezuelan studies [14,55]. This probably is related to the important native American background in Rondônia population.

## Conclusions

In conclusion, genotypes A, D and F found in Rondônia reflecting the ethnic background of its inhabitants, i.e., mainly descendants from European colonizers, African slaves, and indigenous people. Further studies should be carried out to investigate the clinical, virological and therapeutical response characteristics of HBV genotypes, using a large number of samples, including patients representing Rondônia State population with clinical data to characterize their HBV status (carrier, immunotolerancy, acute and chronic hepatitis, cirrhosis and/or hepatocellular carcinoma).

## Competing interests

The authors declare that they have no competing interests.

## Authors' contributions

AOS participated in the design of the study and drafted the manuscript. MVAM conducted the phylogenetic and evolutionary analysis, drafted the manuscript and in its design and coordination. LB participated in the PCR amplification and sequencing process. DSV participated in the design of the study. JRRP participated in the elaboration of the manuscript. FJC, ERH and JMS participated in the design of the study. All authors read and approved the final manuscript.
